# Suppressing Dendrite Growth with Eco-Friendly Sodium Lignosulfonate Additive in Quasi-Solid-State Li Metal Battery

**DOI:** 10.3390/molecules28196905

**Published:** 2023-10-02

**Authors:** Yingkang Tian, Xinyang Chen, Xuejie Gao, Hanyan Wu, Chen Cheng, Shuiping Cai, Wenfeng Ren, Xiaofei Yang, Runcang Sun

**Affiliations:** 1 Center for Lignocellulosic Chemistry and Biomaterials, College of Light Industry and Chemical Engineering, Dalian Polytechnic University, Dalian 116034, China; m15304113180@163.com (Y.T.); cxysmail1@gmail.com (X.C.); wuhy1904@163.com (H.W.); cc15298509429@163.com (C.C.); caishuiping1086@163.com (S.C.); wfren@dlpu.edu.cn (W.R.); 2 Division of Energy Storage, Dalian National Laboratory for Clean Energy, Dalian Institute of Chemical Physics, Chinese Academy of Sciences, 457 Zhongshan Road, Dalian 116023, China; yangxf@dicp.ac.cn

**Keywords:** quasi-solid-state Li metatteries, sodium lignosulfonate, Li dendrite suppression, PVDF-based electrolyte

## Abstract

The application of lithium metal batteries is limited by the drawbacks of safety problems and Li dendrite formation. Quasi-solid-state electrolytes (QSSEs) are the most promising alternatives to commercial liquid electrolytes due to their high safety and great compatibility with electrodes. However, Li dendrite formation and the slow Li^+^ diffusion in QSSEs severely hinder uniform Li deposition, thus leading to Li dendrite growth and short circuits. Herein, an eco-friendly and low-cost sodium lignosulfonate (LSS)-assisted PVDF-based QSSE is proposed to induce uniform Li deposition and inhibit Li dendrite growth. Li symmetric cells with 5%-LSS QSSE possess a high Li^+^ transfer number of 0.79, and they exhibit a long cycle life of 1000 h at a current density/areal capacity of 1 mA cm^−2^/5 mAh cm^−2^. Moreover, due to the fast electrochemical dynamics endowed by the improved compatibility of the electrodes and fast Li^+^ diffusion, the LFP/5%-LSS/Li full cells still maintain a high capacity of 110 mAh g^−1^ after 250 cycles at 6C. This work provides a novel and promising choice that uses eco-friendly LSS as an additive to PVDF-based QSSE in Li metal batteries.

## 1. Introduction

With the rapid development of electrical energy storage devices, traditional lithium-ion batteries (LIBs) are unable to meet the high gravimetric and volumetric energy density demands for energy storage applications such as electric vehicles [1,2,3]. Consequently, lithium metal batteries (LMBs), which utilize lithium metal as an anode material with higher theoretical specific capacity (3860 mAh g^−1^), low gravimetric density (0.534 g cm^−3^), and lower standard potential (−3.04 V vs. the standard hydrogen electrode), have once again captured the attention of researchers [4,5]. However, lithium metal anodes exhibit drawbacks such as high activity, unstable solid electrolyte interphase, the formation of “dead” Li, and dendrite growth, which lead to undesirable electrochemical performance and safety concerns. Recently, quasi-solid-state electrolytes (QSSEs), which present a compromise between liquid electrolytes and solid-state electrolytes [6], have emerged as one of the most promising solutions to address the challenges associated with lithium metal anodes. Typically, QSSEs are created by anchoring the liquid electrolytes with a polymer matrix through physical or electrostatic interactions [1,7]. Compared to conventional liquid electrolytes, QSSEs are less susceptible to electrolyte leakage and combustion. Furthermore, they offer improvements over the drawbacks of poor contact with electrodes and limited ion conductivity (10^−8^–10^−5^ S cm^−1^) at room temperature found in solid polymer electrolytes (SPEs). Therefore, QSSEs are regarded as a promising approach for achieving high-energy-density and high-safety LMBs.

Compared to polyethylene oxide (PEO), poly(vinylidene fluoride) (PVDF) exhibits higher wettability for a liquid electrolyte, better mechanical strength, thermal stability, and more favorable electrochemical stability [8,9]. Although QSSEs and SPEs were initially thought to completely solve the problem of Li dendrite formation due to their high mechanical strength, they still suffer from Li dendrite growth during cycling [10]. Fortunately, adding additives is an effective way to enhance the mechanical strength of the electrolytes and improve battery performance. These additives typically play a crucial role in dendrite suppression and enhancing the ionic conductivity of electrolytes. For instance, Xu et al. [8] hybridized PVDF with a LiF additive to create a composite film, enabling the QSSE with impressive mechanical properties and high ionic conductivity. This combination effectively suppressed Li dendrite and the formation of “dead” Li. Moreover, Rajamani et al. [11] incorporated an LLZTO additive into a PVDF-HFP/PBMA electrospun membrane, increasing the ionic conductivity from 9.924 × 10^−4^ to 4.858 × 10^−3^ S cm^−1^. Additionally, the membrane demonstrated a high electrolyte uptake and porosity, which facilitated rapid Li^+^ migration. Beyond these additives, there is promise in using biomass-based sodium lignosulfonate (LSS) additives to enhance the electrochemical performance of QSSEs. Firstly, lignin is abundant and presents the largest natural resource of aromatic compounds. Its aromatic backbone imparts structural rigidity and thermal stability to lignin-derived materials [12]. Furthermore, research has shown that lignin-based QSSEs contain a substantial number of hydroxyl groups, enabling them to easily form hydrogen bonds with fluorine atoms. These bonds hinder the movement of large anions such as TFSI^−^ and promote Li^+^ transport [13]. Moreover, the hydrogen bonds between the liquid electrolyte and lignin result in a high strength and superior comprehensive electrochemical performance [14]. In addition, lignosulfonate features sulfonic groups. These sulfonate groups with high electronegativity simultaneously facilitate electrolyte access, promote ion pair dissociation and increase Li^+^ mobility [15]. They also exhibit an excellent SEI-forming ability. For example, Xu et al. [16] designed a sulfonate-rich covalent organic framework (named SCOF-2)-modified separator, which possessed strong electronegativity, endowing the separator with higher Li^+^ transfer number and better Li dendrite suppression compared to sulfonate-free COFs. Liu et al. [17] developed a sodium lignosulfonate (LSS)/PEO composite electrolyte and demonstrated strong capability in Li dendrite suppression. Therefore, it is promising and effective to use LSS as an additive to suppress Li dendrite in LMBs.

Herein, we designed an environmentally friendly PVDF-based QSSE with an optimized 5 wt.% LSS additive (labeled as 5%-LSS) via a combination of solution casting and a phase-inversion freeze-drying method [18]. And the QSSE without LSS named bare QSSE. For 5%-LSS QSSE, the abundant hydroxyl groups in the LSS hindered the transfer of large anions such as TFSI^-^ in the electrolyte by forming hydrogen bonds with fluorine atoms. Additionally, the sulfonate groups in LSS served as affinity sites for transporting Li^+^, enhancing uniform Li deposition [19,20]. These characteristics endowed the 5%-LSS QSSE with a high Li^+^ transfer number of 0.79 and excellent Li dendrite suppression capability. The assembled Li-Li symmetric cells stably run for over 300 h and 1000 h at the current densities/areal capacities of 1 mA cm^−2^/1 mAh cm^−2^ and 1 mA cm^−2^/5 mAh cm^−2^, respectively. In addition, the LiFePO_4_ (LFP)/5%-LSS QSSE/Li full cells exhibited a high capacity of 110.5 mAh g^−1^ after 250 cycles under a high rate of 6C. 

## 2. Results and Discussion

### 2.1. Materials Characterization

**Figure 1:**
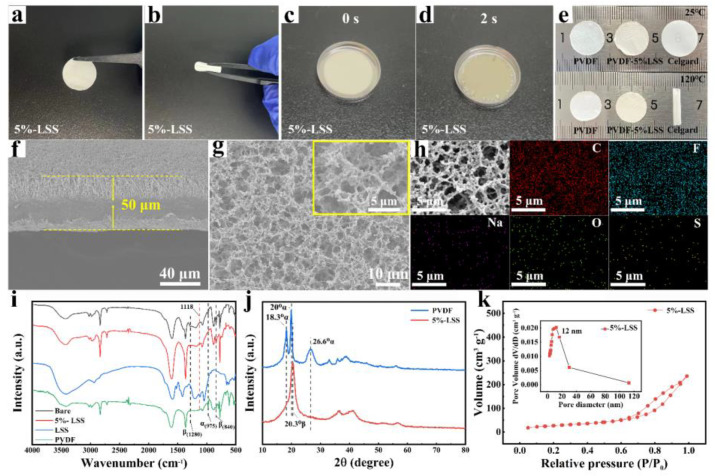
Characteristic of the samples. (**a**–**d**) Optical images of the 5%-LSS membranes. (**e**) Dimensional stability test of different membranes. (**f**) The cross-section image of the 5%-LSS membrane. (**g**,**h**) SEM image and corresponding mapping results of the 5%-LSS membranes. (**i**) The FTIR results of the bare membrane, 5%-LSS membrane, LSS powder and PVDF powder. (**j**) XRD results of the PVDF power and the 5%-LSS membrane. (**k**) BET results of the 5%-LSS membrane.

**Figure 2:**
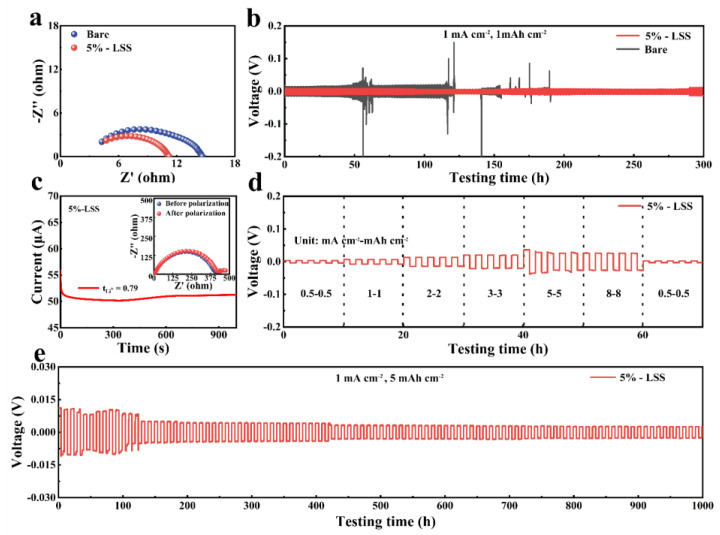
Electrochemical performance of 5%-LSS QSSE. (**a**) EIS plot of Li symmetric cells with 5%-LSS QSSE and bare QSSE after 5 cycles. (**b**) Li symmetric cells with different QSSEs cycled at a current density/areal capacity of 1 mA cm^−2^/1 mAh cm^−2^. (**c**) I-t curve and EIS plots before and after polarization for 5%-LSS QSSE. (**d**) Rate capability testing of 5%-LSS symmetric cell at current densities from 0.5 to 8 mA cm^−2^. (**e**) 5%-LSS Li symmetric cell cycling at a current density/areal capacity of 1 mA cm^−2^/5 mAh cm^−2^.

The additive-free PVDF membrane appeared to have a white color, as shown in Figure S1, while the 5%-LSS membrane seemed to have a slight brown tint after the addition of the LSS additive, as displayed in Figure 1a. In addition, the membrane could be freely bent (Figure 1b), indicating its good flexibility. The wettability of the 5%-LSS membrane is shown in Figure 1c,d, where it could be observed that the gel process was completed within two seconds after adding 50 μL electrolyte, indicating the superior wettability, which is helpful for achieving a high ionic conductivity [21]. The good wettability should be attributed to the high porosity of the 5%-LSS membrane. As the scanning electron microscope (SEM) images shown in Figure 1g and Brunauer–Emmett–Teller (BET) surface area analysis results in Figure 1k indicate, the 5%-LSS membrane revealed a porous structure. And the N_2_ adsorption–desorption isotherms of 5%-LSS exhibited type IV with an obvious H3 hysteresis loop, indicating the distribution of mesoporous channels in the samples [22,23,24]. Compared to the pore size distribution curve (Figure S5) of bare membranes, 5%-LSS membranes featured a more uniform pore size distribution in the range of 2–30 nm. The porous structure and uniform pore size distribution endowed 5%-LSS membranes with high electrolyte uptake [25] and suitable transport pathways for fast Li^+^ transfer [26], respectively. These properties mattered considerably in the performance of cells. As shown in Figure 1f, the 5%-LSS membrane had a thickness of 50 μm, which is similar to that of the bare membrane (52 μm) shown in Figure S2. Thermal stability was also considered a critical parameter of cells, and that of different membranes was demonstrated in Figure 2e. The commercial Celgard2400 separator shrank severely when exposed to 120 °C for 30 min, while the bare membrane and 5%-LSS membrane only experienced a slight decrease in dimension under the same conditions, proving the high thermal stability of a PVDF substrate. The elemental mapping images (Figure 1h) showed that LSS additives containing elements of Na, O, and S were uniformly dispersed in the membrane, which was beneficial for Li^+^ transfer. In comparison, the bare one (Figure S3) only involved the elements of C and F. The Fourier-transform infrared spectroscopy (FTIR, Figure 1i) spectrum further demonstrated the successful addition of LSS. After the introduction of LSS, an absorption band at approximately 1120 cm^−1^ appeared in the spectrum of 5%-LSS, indicating the existence of sulfonic groups on the surface of the 5%-LSS membrane [27]. In addition, the membranes prepared using the phase-inversion method exhibited a vibrational peak in the FTIR spectrum. The bare membranes spectrum showed intensive absorption bands at 840 and 1280 cm^−1^ (characteristics of the β phase) with a weak peak at 975 cm^−1^ (characteristic of the α phase) [28]. On the contrary, the PVDF powder, dominated by the α phase, exhibited an opposite result, suggesting that the phase-inversion method successfully prepared β-phase-dominated membranes. This phenomenon could be explained by the stretching between segments of polymer chains after dissolution and recrystallization during phase inversion, which might contribute to reducing the crystallinity of PVDF [29]. In addition, it further enhanced the porosity and electrolyte uptake of the membranes, creating a uniform ion transport pathway [30,31]. Moreover, the polar phase (β phase) membranes also possessed better electrochemical stability [25]. As shown in the XRD characterization (Figure 1j), there were two intense diffraction peaks at 18.3° and 20° as well as a medium peak of 26.6°, corresponding to the (020), (110) and (021) planes of α-PVDF. In contrast, the 5%-LSS membrane was dominated by the β phase, which exhibited intense diffraction at 20.3°, reflecting the (110/220) planes of β-PVDF [32]. Additionally, it was also true for the XRD characterization of bare membranes (Figure S4), suggesting that the addition of LSS had a negligible effect on the phase conversion of PVDF. Overall, these results further demonstrate the successful phase inversion of PVDF polymers from the α phase to the β phase.

### 2.2. Li Symmetric Cells with Different QSSEs

The interfacial properties between Li metal and QSSE were investigated by using electrochemical impedance spectroscopy (EIS) after 5 cycles at a current density/areal capacity of 1 mA cm^−2^/1 mAh cm^−2^ (Figure 2a). The semicircle at the high-frequency region reflected the charge transfer resistance (R_ct_). The results showed that the R_ct_ of Li symmetric cells with 5%-LSS QSSE and bare QSSE after 5 cycles were 11 Ω and 14 Ω, respectively. The smaller R_ct_ of the cell using 5%-LSS QSSE indicated better electrochemical dynamics. The improvement could be attributed to the better contact between 5%-LSS QSSE and the electrode, which is beneficial for fast Li^+^ diffusion and induces uniform Li deposition [33,34,35]. As a proof of the concept, the Li dendrite suppression capability was evaluated by the galvanostatic cycling test of Li symmetric cells at a current density of 1 mA cm^−2^ and an areal capacity of 1 mAh cm^−2^. As shown in Figure 2b and Figure S6, both cells exhibited excellent cycling stability in the first 25 h with an overpotential of 8 mV (5%-LSS QSSE) and 18 mV (bare QSSE), respectively. However, an increase in overpotential after 50 h and a complete short circuit after 100 h is observed [36], suggesting the poor capability of bare QSSE in Li dendrite suppression. On the contrary, the cells assembled with the 5%-LSS QSSE remained stable with an overpotential of roughly 10 mV in 300 h, indicating the improved Li dendrite suppression capability within the introduction of the LSS additive. To characterize the Li^+^ migration ability in 5%-LSS QSSE, the Li^+^ transference number ($t_{{Li}^{+}}$) was measured using I-T curves and EIS measurements. Figure 2c shows the typical current–time curve of DC polarization obtained by 5%-LSS QSSE. The values for the I_0_ and I_s_ were 55.89 μA and 51.23 μA, respectively. The inset graph in Figure 2c is the EIS plot depicting the values of R_0_ and R_s_ to be 400.5 Ω and 413.7 Ω, respectively. According to the above data, the $t_{{Li}^{+}}$ was calculated to be 0.79, which is higher than most reported liquid electrolytes [36]. A larger $t_{{Li}^{+}}$ indicates less concentration polarization during the cell charging and discharging, resulting in reduced electrode overpotential and bulk impedance [37], as reflected in the voltage profile in Figure 2b. Furthermore, the rate capability of 5%-LSS QSSE was evaluated at various current densities ranging from 0.5 to 8 mA cm^−2^ (half cycling time: 1 h). As shown in Figure 2d, the cell using 5%-LSS QSSE exhibited a low overpotential of 3.3 mV at a current density of 0.5 mA cm^−2^. Even at a high current density of 8 mA cm^−2^, it exhibited an overpotential value of 26 mV, indicating stable Li plating and stripping behavior. Upon returning to a current density of 0.5 mA cm^−2^, the overpotential recovered to a low value of 3 mV, indicating the excellent reversibility of the symmetric cell with 5%-LSS QSSE. In addition, a cell with 5%-LSS QSSE under elevated areal capacity (5 mAh cm^−2^) at a current density of 1 mA cm^−2^ was also tested. The high areal capacity was found to be closely related to the plating/stripping behavior of the Li anode surface [38]. As shown in Figure 2e, during the first 120 h, the voltage profile exhibited an overpotential of around 11 mV. For the next 880 h, possibly due to the electronegative sulfonate group interacting with a TFSI^-^ anion to promote Li^+^ transfer and form uniform Li deposition, the cell’s overpotential was stabilized at 3 mV, further indicating the excellent electrochemical compatibility of the 5%-LSS QSSE with the Li anode [35] and its superior ability to suppress lithium dendrite.

**Figure 3:**
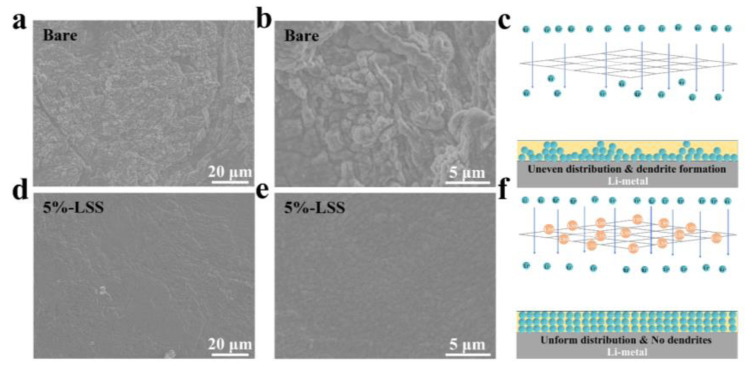
The top view of SEM images of plated Li anode by using different QSSEs. (**a**,**b**) The bare QSSE. (**d**,**e**) 5%-LSS QSSE. Schematic illustration of cycled Li behaviors with (**c**) Bare QSSE (**f**) 5%-LSS QSSE.

To further analyze the effect of 5%-LSS QSSE on Li deposition in the cell, the morphology of the Li anode after the first plating was checked by SEM. As shown in Figure 3a,b, the Li anode using bare QSSE exhibited an uneven morphology and dead Li was observed, which confirmed the uneven Li deposition and coincided well with the electrochemical performance of the Li symmetric cell [20,39]. As illustrated in Figure 3c, during the Li^+^ plating process, the Li^+^ flux became non-uniform after passing through the bare membrane, resulting in uneven Li distribution and the formation of a Li dendrite. In contrast, the Li anode presented a smooth surface without any noticeable Li agglomerations in the presence of 5%-LSS QSSE (Figure 3d), which was also confirmed in the locally enlarged image (Figure 3e). In addition, as illustrated in Figure 3f, the reactive sulfonate groups [17,20] and the abundance of hydroxyl groups in LSS [13] contributed to the stable and fast Li^+^ transport, improving the Li^+^ dissociation process when LSS molecules were combined with PVDF molecules [40]. This, in turn, resulted in uniform Li deposition and a significant improvement in the cycling life of the cells.

### 2.3. Li Full Cells with Different QSSEs

**Figure 4:**
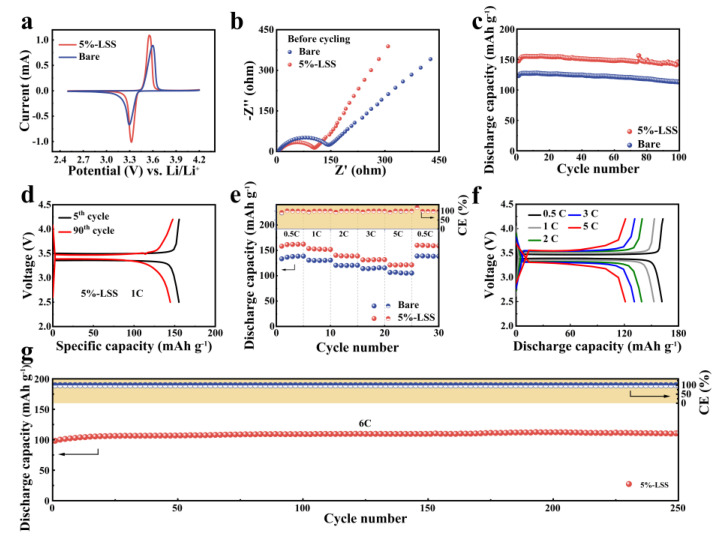
Electrochemical performance of LFP (LiFePO_4_) full cells with bare and 5%-LSS QSSE. (**a**) Cyclic voltammetry (CV) curves. (**b**) EIS plots before cycling. (**c**) Cycling performance at 1C. (**d**) Charge/discharge curves of the cell using 5%-LSS QSSE at 1C. (**e**) Rate performance of different cells from 0.5 to 5C. (**f**) The corresponding charge/discharge performance of the cell using 5%-LSS QSSE. (**g**) Cycling performance of the cell using 5%-LSS QSSE at 6C.

Meanwhile, the LFP full cells were used to further investigate the electrochemical performance of 5%-LSS QSSE using an LFP cathode and Li metal anode. The cyclic voltammetry curves were obtained at a scan rate of 0.1 mV s^−1^, as shown in Figure 4a. The oxidation and reduction peaks of LFP in the cell using bare QSSE were located at 3.60 and 3.30 V (vs. Li^+^/Li), respectively, with a potential difference (ΔV) of 0.3 V. On the contrary, the cell with 5%-LSS QSSE showed a smaller ΔV of approximately 0.24 V, which was calculated based on the value of the oxidation peak (3.56 V) and reduction peak (3.32 V). The current intensity was higher at both peaks, indicating that the LSS allowed for faster electrochemical reaction kinetics [41]. The result was consistent with the EIS plot (Figure 4b) before cycling, which consisted of two parts: a depressed semicircle in the high-frequency region and a sloping line in the low-frequency region, reflecting the R_ct_ and the resistance of Li^+^ diffusion. The cell using 5%-LSS QSSE exhibited a lower R_ct_ of 104 Ω compared to the bare QSSE (144 Ω). These results confirmed that the LSS additive significantly enhanced the electrochemical reaction kinetics [13,42]. Furthermore, the cycling stability and rate performance of the full cells were tested. As shown in Figure 4c, in the 5th cycle, the discharge capacities of the 5%-LSS QSSE and bare QSSE-based cells were 155.2 mAh g^−1^ and 127.4 mAh g^−1^, respectively. After 90 cycles, the cell with 5%-LSS QSSE still exhibited a discharge capacity of 144.6 mAh g^−1^, which was higher than the bare cell (115.2 mAh g^−1^). The discharge capacity retention of the 5%-LSS QSSE-based cell was 93.2%, which was higher than the bare QSSE (90.4%). The superior capacity retention of the 5%-LSS QSSE-based cell could also be confirmed by the charging–discharging profiles in Figure 4d and Figure S7. Rate performance is another important parameter to evaluate the fast charging/discharging and reversibility ability of a cell [35]. As shown in Figure 4e, the rate performance of 5%-LSS QSSE and bare QSSE-based cells under various C rates from 0.5 to 5C was also studied. The high discharge capacities of 161, 152.7, 139.1, 131, and 121 mAh g^−1^ were achieved by the cell with 5%-LSS QSSE at the rates of 0.5, 1, 2, 3, and 5C, respectively, while the cells using bare QSSE delivered discharge capacities of 136, 130, 120, 113.9, and 106 mAh g^−1^, respectively. When the C rate returned to 0.5C, the 5%-LSS cell recovered its high capacity to 159 mAh g^−1^, indicating excellent reversibility after cycling under high current densities. Moreover, as shown in Figure 4f, the overpotentials were less than 0.13 V under all C rates, demonstrating the good charge/discharge performance and excellent electrochemical kinetics endowed by the LSS additive [41,42]. Furthermore, a full cell at 6C is also evaluated. As shown in Figure 4g, the cell assembled with 5%-LSS QSSE demonstrated a high capacity of roughly 110 mAh g^−1^ for over 250 cycles, revealing its potential application in fast-charging cells [43]. In this regard, LFP full cells containing lignin molecules and sulfonate acid groups presented improved cycling performance as well as excellent rate capability.

### 2.4. Schematic

**Figure 5:**
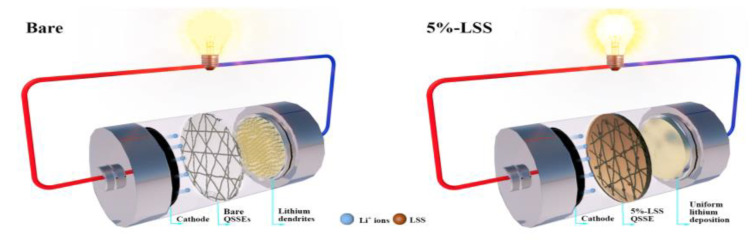
Schematic illustration of the lithium deposition of PVDF-based QSSEs without and with the 5%-LSS additive on the Li anode.

As shown in Figure 5, the different QSSEs in the cell led to various Li surface morphologies after cycling. The addition of 5%-LSS to PVDF-based QSSE resulted in the presence of hydroxyl and sulfonate groups, leading to uniform lithium deposition [13,20]. Additionally, the existence of hydrogen bonds between the LSS and organic liquid electrolyte also contributed to excellent mechanical strength and superior comprehensive electrochemical performance [14]. Consequently, this created a smooth surface on the Li anode. In this case, the 5%-LSS QSSE endowed the cell with uniform Li deposition, showcasing the capability of the LSS-assisted PVDF-based QSSE in suppressing Li dendrite. In contrast, the cell with bare QSSE suffered from random Li^+^ flux, resulting in the random nucleation of lithium on the anode surface. With continued cycling, uncontrolled dendrite growth and dead Li formation could lead to cell performance degradation, short circuits, and even safety issues.

## 3. Experimental

### 3.1. Materials

PVDF powder was purchased from Hefei Ke Jing Materials Technology Co., Ltd. (Hefei, China) Dimethyl sulfoxide (DMSO) was purchased from Tianjin Kermel Chemical Technology Co., Ltd. (Tianjin, China) Sodium lignosulfonate (LSS) was purchased from Shanghai Macklin Biochemical Technology Co., Ltd. (Shanghai, China) The liquid electrolyte (1.0 M LiTFSI in DOL: DME = 1:1 Vol%, with 1.0% LiNO_3_) was supplied by Guangdong Canrd New Energy Technology Co., Ltd. China (Dongguan, China).

### 3.2. Synthesis of PVDF Membranes

The PVDF membranes were fabricated using the typical doctor-blade method. Firstly, the PVDF powder was dissolved in DMSO with a concentration of 9 wt.%, which was followed by stirring it for 72 h at 60 °C. After that, the precursor solution was cast on the glass plate with a scraper gap of 400 μm and left to air dry for 2 h to form PVDF membranes. Subsequently, the membranes were immersed in deionized water for phase-inversion solution replacement. Finally, the hydrogel was dried in a freeze-dryer for 24 h to remove residual water and obtain the PVDF polymer film that called bare membrane.

### 3.3. Synthesis of PVDF-5%-LSS Membranes

PVDF powder and sodium lignosulfonate in the mass ratio of 95:5 were dissolved in the above solution and labeled as 5%-LSS membrane. The subsequent steps were the same as before. The thickness of the 5%-LSS membrane was measured using an SEM cross-section image, which showed 50 μm.

### 3.4. Synthesis of Quasi-Solid-State Electrolytes

The QSSEs were obtained by dropping 50 μL of liquid electrolyte (1.0 M LiTFSI in DOL: DME = 1:1 Vol%, with 1.0% LiNO_3_) into the membranes with 15 mm diameters in an argon-filled glove box (water content < 0.01 ppm, oxygen content < 0.01 ppm) for further measurements.

### 3.5. Materials Characterizations

The morphology and structure of membranes and the Li anode surface were characterized using scanning electron microscopy (SEM) (Hitachi S-4800, Tokyo, Japan) that was equipped with an energy-dispersive spectrometer (EDS). The dimensional stability test of the different separators was conducted in the blast-drying oven at a temperature of 120 °C for 30 min. The Fourier transform infrared (FT-IR) spectra were measured in the range of 450–4000 cm^−1^ with a resolution of 4 cm^−1^ using an FT-IR Spectrometer (Spectrum Two, Waltham, MA, USA). The PVDF powder and different membranes were investigated at a scan rate of 2 min^−1^ from 10° to 80° by X-ray diffraction (SHIMADZU XRD-6100, Kyoto, Japan). The pore size and distribution of bare membranes and 5%-LSS membranes were tested using Brunauer–Emmett–Teller (Kubo-X1000, Beijing, China) at a drying temperature of 45 °C for 6 h. All of the visual images were taken by phone camera.

### 3.6. Electrochemical Measurement

The Li symmetric cells and LFP/QSSE/Li full cells were assembled with the standard of CR2023 cion-type cell configurations in an argon-filled glove box (water content < 0.01 ppm, oxygen content < 0.01 ppm). The synthesized QSSE were sandwiched between the two electrodes of cell. For the LFP (LiFePO_4_) cathode, it was prepared by pasting a slurry (80 wt.% LFP powder, 15 wt.% Super P and 5 wt.% PVDF as binder) using N-methylpyrrolidone as solvent onto double-sided carbon-coated aluminum foil, which was then vacuum dried at 60 °C for 7 h. All of the cells were charged and discharged using the LANDMon system.

The QSSE’s impedance was tested on the electrochemical workstation (CHI660E, Shanghai Chenhua Co., Ltd., Shanghai, China) using electrochemical impedance spectroscopy (EIS) with the Li symmetric cells. 

The Li^+^ transfer number was also conducted on the electrochemical workstation. The I-t curves were tested for the sensitivity of 1 × 10^−4^ A V^-1^ at a chronoamperometry potential of 30 mV for 1000 s using Li symmetric cells. The value was calculated using the following equation:

(1) $$t_{{Li}^{+}}=\frac{I_{s}(V-I_{0}R_{0})}{I_{0}(V-I_{s}R_{s})}$$

where $t_{{Li}^{+}}$ is the transfer number, and *I_s_* and *I*_0_ represent the current at the steady state and initial state, respectively. *R*_0_ and *R_s_* are the internal resistances in their respective states: initial and steady state. In this work, the initial voltage is 30 mV.

The cyclic voltammetry (CV) and impedance spectra were also tested on the above workstation using LFP/QSSE/Li cells within the potential range of 2.5 to 4.2 V. Additionally, the scan rate and sensitivity of the CV test were 1 × 10^−4^ V s^−1^ and 1 × 10^−3^ A V^−1^, respectively.

## 4. Conclusions

In summary, an LSS additive-assisted PVDF-based QSSE is developed to induce uniform Li nucleation and smooth Li deposition, leading to assembled cells with prolonged cycling, excellent rate performance and improved safety. The lignin molecule and sulfonate acid groups with high electronegativity simultaneously facilitate electrolyte access, promote ion pair dissociation and increase Li^+^ mobility, thus increasing the Li^+^ transfer number and improved ionic conductivity. As a result, the Li symmetric cell using 5%-LSS QSSE achieved a long cycle life of 300 h at a current density/areal capacity of 1 mA cm^−2^/1 mAh cm^−2^ and high Li^+^ transfer number of 0.79. Moreover, the symmetric cell also demonstrates an ultra-long-life of 1000 h at a current density/areal capacity of 1 mA cm^−2^/5 mAh cm^−2^, indicating that the cell assembled with 5%-LSS QSSE possesses a superior capability to suppress Li dendrites. Additionally, the addition of 5%-LSS significantly enhances the electrochemical performance of PVDF-based QSSE in LFP full cells. As a result, the LFP/5%-LSS QSSE /Li full cell exhibited smaller potential difference (0.24 V) than that of the bare QSSE (0.3 V) in a CV test. Furthermore, the LFP/5%-LSS/Li full cells exhibit a high capacity of 110.5 mAh g^−1^ after 250 cycles at 6C, owing to the fast Li^+^ diffusion and excellent compatibility between 5%-LSS QSSE and electrodes. Therefore, it is believed that the LSS is a promising additive for low-cost and eco-friendly quasi-solid-state Li metal batteries.

## Data Availability

The data presented in this study are available in the Supplementary Materials.

## Supplementary Materials

The following supporting information can be downloaded at https://www.mdpi.com/article/10.3390/molecules28196905/s1, Figure S1: Optical image of the bare membrane. Figure S2: Cross-section SEM image of the bare membrane. Figure S3: Mapping images of bare membrane. Figure S4: XRD characterization of PVDF powder and bare membrane. Figure S5: The pore size and pore distribution of the membranes. Figure S6: Amplified Li symmetric cell with 5%-LSS QSSE and bare QSSE cycled at current density/ areal capacity of 1 mA cm^−2^/1 mAh cm^−2^ for different cycles: (a) the 25th cycle, (b) the 50th cycle, (c) the 100th cycle, (d) the 150th cycle. Figure S7: The charge and discharge curves of LFP/bare QSSE/Li at the 5th and 90th cycles.
