# Supplementary material for: Suppressing Dendrite Growth with Eco-Friendly Sodium Lignosulfonate Additive in Quasi-Solid-State Li Metal Battery
Source: Molecules. 2023 Oct 2;28(19):6905. doi: 10.3390/molecules28196905 (PMC10574181; doi:10.3390/molecules28196905)
Supplement: Supplementary file 1 [file molecules-28-06905-s001.zip › molecules-2535263-supplementary.pdf]

Supporting Information

Eco-friendly Sodium Lignosulfonate Additive assisted PVDF-based Quasi-solid-state Electrolyte for Dendrite-free High performance Li Metal Battery

Yingkang Tian[1,#], Xinyang Chen[1,#], Xuejie Gao[1,*], Hanyan Wu[1], Chen Cheng[1], Shuiping Cai[1], Wenfeng Ren[1], Xiaofei Yang[2], Runcang Sun[1,*]

[1] *Center for Lignocellulosic Chemistry and Biomaterials, College of Light Industry and Chemical Engineering, Dalian Polytechnic University, Dalian, 116034, China*
[2]*Division of Energy Storage, Dalian National Laboratory for Clean Energy, Dalian Institute of Chemical Physics, Chinese Academy of Sciences, 457 Zhongshan Road, Dalian 116023, China*
*Corresponding Author: gaoxuejie1107@163.com (X. Gao), rcsun3@dlpu.edu.cn (R. Sun)

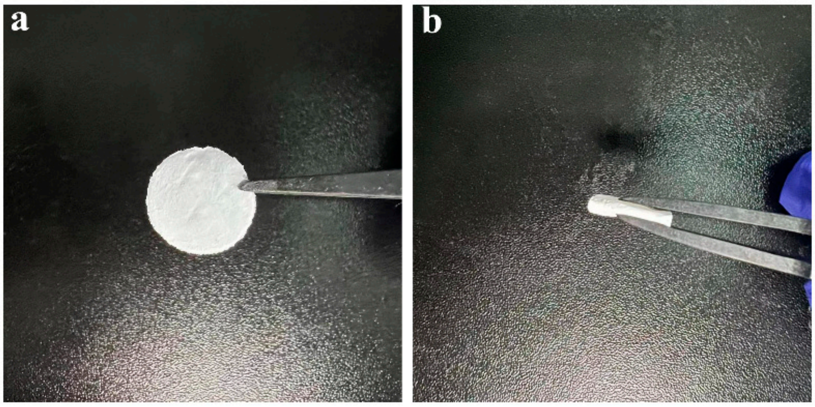

Figure S1. Optical image of the bare PVDF membrane.

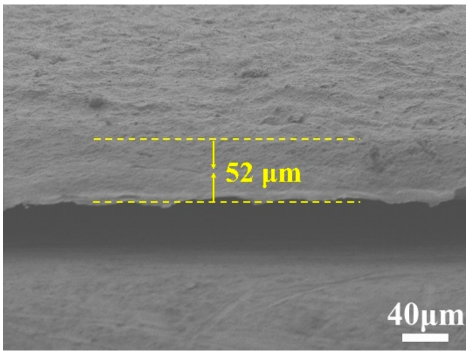

Figure S2. Cross-section SEM image of bare PVDF membrane.

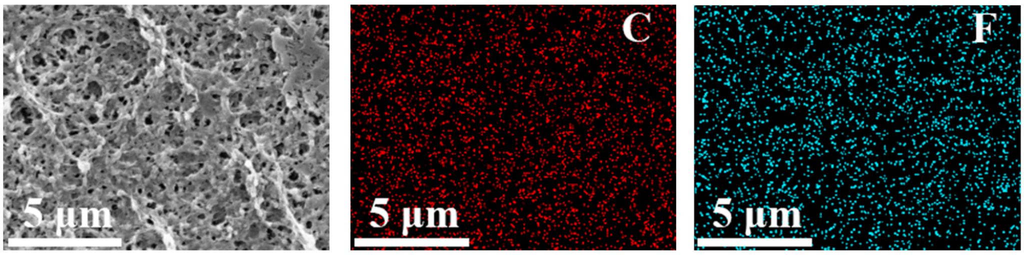

Figure S3. Mapping images of pure PVDF membrane.

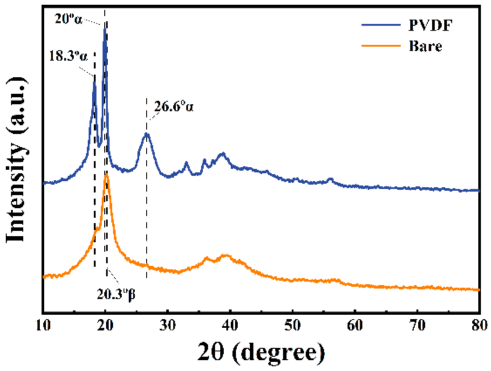

Figure S4. XRD characterization of PVDF powder and Bare

membrane

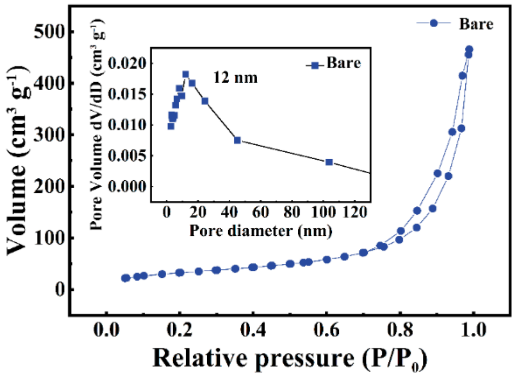

Figure S5. The pore size and pore distribution of the PVDF membranes.

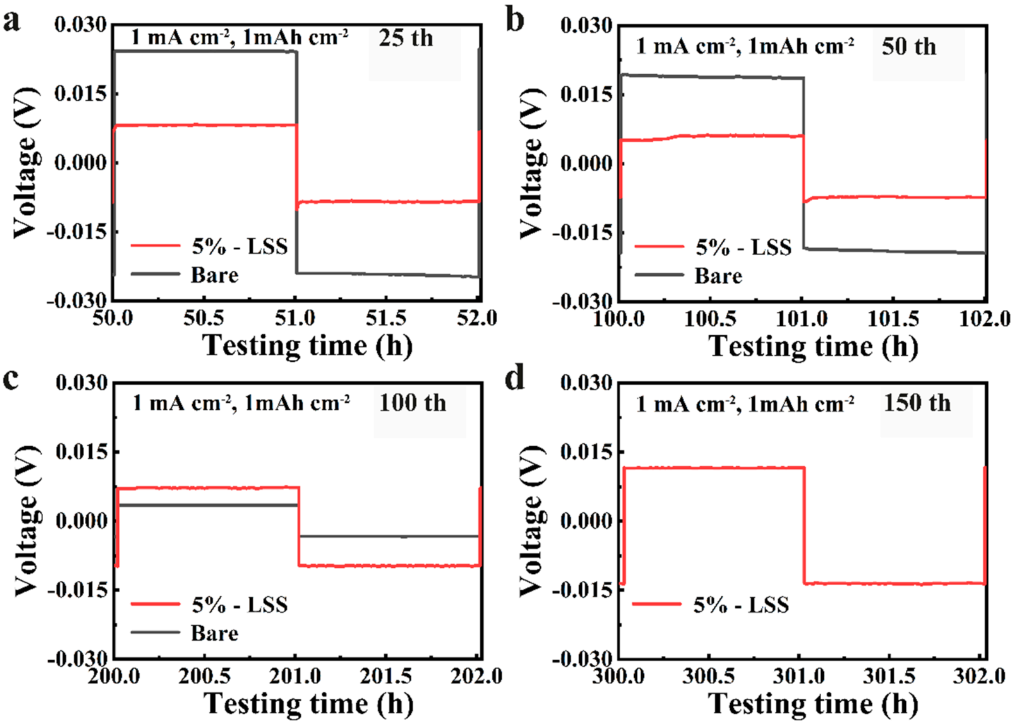

Figure S6. Amplified Li symmetric cell with 5%-LSS-PVDF QSSEs and Bare QSSEs at 1mA cm^{-2} - 1mAh cm^{-2} of (a) 25th cycle (b) 50th (c) 100th (d) 150th.

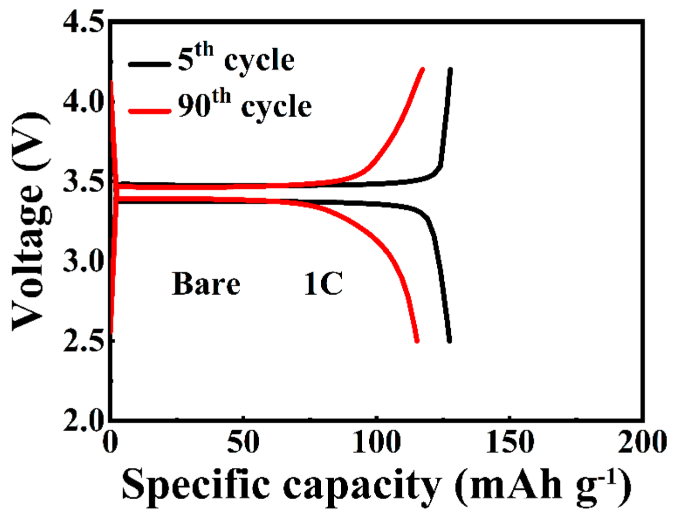

Figure S7. The charge and discharge curve of LFP/PVDF-QSSEs/Li at the 5th and 90th.
